# Persistent Infection by *Wolbachia w*AlbB Has No Effect on Composition of the Gut Microbiota in Adult Female *Anopheles stephensi*

**DOI:** 10.3389/fmicb.2016.01485

**Published:** 2016-09-21

**Authors:** Shicheng Chen, Jiangchao Zhao, Deepak Joshi, Zhiyong Xi, Beth Norman, Edward D. Walker

**Affiliations:** ^1^Department of Microbiology and Molecular Genetics, Michigan State University, East LansingMI, USA; ^2^Department of Animal Science, University of Arkansas, FayettevilleAR, USA; ^3^Department of Entomology, Michigan State University, East LansingMI, USA

**Keywords:** *Anopheles*, *Wolbachia*, malaria vectors, paratransgenesis

## Abstract

The bacteria in the midgut of *Anopheles stephensi* adult females from laboratory colonies were studied by sequencing the V4 region of 16S rRNA genes, with respect to three experimental factors: stable or cured *Wolbachia* infection; sugar or blood diet; and age. *Proteobacteria* and *Bacteroidetes* dominated the community [>90% of operational taxonomic units (OTUs)]; most taxa were in the classes *Flavobacteriia*, *Gammaproteobacteria*, and *Alphaproteobacteria*, and were assigned to *Elizabethkingia* (46.9%), *Asaia* (6.4%) and *Pseudomonas* (6.0%), or unclassified *Enterobacteriaceae* (37.2%). Bacterial communities were similar between *Wolbachia*-cured and *Wolbachia*-infected mosquito lines, indicating that the gut microbiota were not dysregulated in the presence of *Wolbachia*. The proportion of *Enterobacteriaceae* was higher in mosquitoes fed a blood meal compared to those provided a sugar meal. Collectively, the bacterial community had a similar structure in older *Wolbachia*-infected mosquitoes 8 days after the blood meal, as in younger *Wolbachia*-infected mosquitoes before a blood meal, except that older mosquitoes had a higher proportion of *Enterobacteriaceae* and lower proportion of *Elizabethkingia*. Consistent presence of certain predominant bacteria (*Elizabethkingia*, *Asaia*, *Pseudomonas*, and *Enterobacteriaceae*) suggests they would be useful for paratransgenesis to control malaria infection, particularly when coupled to a *Wolbachia*-based intervention strategy.

## Introduction

Strategies for controlling mosquito-vectored pathogens based on infection with the symbiotic bacterium *Wolbachia* include vector population replacement and population suppression (Sinkins, 2004; Werren et al., 2008; Iturbe-Ormaetxe et al., 2011; Bourtzis et al., 2014). Although many mosquito species are naturally infected with various strains of *Wolbachia*, artificially but stably induced *Wolbachia* infection in mosquito host species not naturally having *Wolbachia* infection was originally established by embryonic injection followed by *trans*-generational spread (Dobson et al., 1999; Sinkins, 2004; Bourtzis et al., 2014). Certain combinations of *Wolbachia* strains and mosquito species suppress propagation and transmission of dengue viruses and malaria parasites in mosquito hosts (Bourtzis et al., 2014). However, in nature the host range of *Wolbachia* does not include some of the most important vectors of human pathogens, such as *Anopheles stephensi, A. gambiae*, and *Aedes aegypti* (Hilgenboecker et al., 2008). This host range has been expanded through forced infections by inoculations into mosquito embryos, resulting in stable infection across subsequent generations in *A. aegypti* and *A. stephensi* with little fitness cost (Xi et al., 2005; Bian et al., 2013; Joshi et al., 2014). A recent study found strongly negative interactions between the commensal gut microflora in mosquitoes and propensity of the mosquitoes to harbor *Wolbachia* infection after intrathoracic inoculation, which might explain why natural infections of *Wolbachia* in some mosquito species are not found in nature (Hughes et al., 2014). Hughes et al. (2014) reported that *Wolbachia* vertical transmission was achievable in *Anopheles* species only when the gut microbiota (especially, *Asaia* bacteria) were perturbed (i.e., eliminated) with antibiotics. Furthermore, Rossi et al. (2015) observed that *Wolbachia* failed to colonize the female reproductive organs of anophelines, due to presence of *Asaia* infection in those tissues (Rossi et al., 2015). The conclusion of both studies is that commensal microbiota impede *Wolbachia* vertical transmission in mosquitoes (Hughes et al., 2014; Rossi et al., 2015). However, what remains to be determined is whether the commensal microbiota of mosquitoes that have been stably infected *trans*-generationally with *Wolbachia* by embryonic injection differ from that of *Wolbachia*-uninfected mosquitoes of the same species and held under the same conditions. Based on the results of studies reviewed here (Hughes et al., 2014; Rossi et al., 2015), one would predict that stably infected mosquitoes must differ in their microbiota due to the inhibitory effects of *Asaia* and other gut symbionts on *Wolbachia* infection. Because stably infected mosquitoes are those most likely to be used in attempts to control vector populations or pathogen transmission, the interactions between stable *Wolbachia* infection and gut microbiota clearly become important.

After the *w*AlbB *Wolbachia* strain was successfully transferred from *A. albopictus* into *A. stephensi* by embryonic microinjection, this strain of *Wolbachia* spread rapidly into a laboratory population of *A. stephensi* by vertical transmission over the course of several generations without any manipulation of gut microbiota (Bian et al., 2013). Further, *w*AlbB infection conferred partial resistance to development of the malaria parasite *Plasmodium falciparum* in *A. stephensi* (Bian et al., 2013), and significantly up-regulated the mosquitoes’ innate immune response, including generation of reactive oxygen species from midgut epithelial cells (Pan et al., 2012). One way to increase the inhibitory effect of *Wolbachia* on virus or parasite development in mosquito hosts is to increase the intensity of *Wolbachia* infection (Correa and Ballard, 2014). Another strategy is to couple paratransgenesis of gut microbiota with a *Wolbachia*-based vector population replacement/suppression strategy. The latter concept is promising because certain symbionts such as *Asaia* and *Pantoea agglomerans*, when engineered to express molecules having anti-malaria parasite properties, result in reduced malaria parasite load in host mosquitoes (Wang et al., 2012; Bongio and Lampe, 2015). However, the innate immune response triggered by *Wolbachia* infection is non-specific and it may affect the commensal microflora (Pan et al., 2012). Thus, it remains unclear if introduced or genetically modified bacteria will persist in *Wolbachia*-infected mosquitoes due to elevated innate immune response and putative incompatibility between *Wolbachia* and gut microbiota. If *Wolbachia w*AlbB infection with its non-specific immune effects in *A. stephensi*, and engineered gut microbiota with specific anti-parasite effects, are found to be compatible; then a synergistic, dual strategy could be developed to suppress malaria parasite or virus development in mosquitoes. In this study, and as a step toward determining the extent of this compatibility, we quantified the diversity of the bacterial community in stably *Wolbachia*-infected and *Wolbachia-*cured *A. stephensi* mosquitoes. Moreover, because of the dynamics of microbiota associated with key life history events in mosquitoes, we further compared bacterial communities when sugar or meals were provided, and when the mosquitoes were young or aged.

## Materials and Methods

### Ethics Statement

All procedures involving vertebrate animals were in accordance with the recommendations in the Guide for the Care and Use of Laboratory Animals of the National Institutes of Health, and were conducted under protocol 03/14-036-00, approved by the Michigan State University Institutional Animal Care and Use Committee.

### Insects

Mosquito colonization procedures are described elsewhere (Chen et al., 2015). Briefly, adult mosquitoes were held in cages at 27°C and 85% humidity under a light/dark 12:12-h photoperiod without dawn/dusk transitions, and were provided a 10% sucrose solution *ad libitum* by cotton wick. To initiate egg development, bovine blood with sodium heparin (Hemostat Lab, Dixon, CA, USA) was fed to adult mosquitoes via an artificial membrane feeder. Two days after the blood meal, oviposition substrates (consisting of filter paper moistened with water in a Petri dish) were placed in the cages. Eggs were transferred to plastic containers with distilled water for hatching. First Bite (Kyorin, Himeji, Japan) and Tetramin tropical fish food flakes (Tetra, Blacksburg, VA, USA) were provided *ad libitum* to first instar larval mosquitoes. Thereafter, pet food (Purina Cat Chow; Nestleé) was given once per day *ad libitum*.

The stable *w*AlbB infection in the *A. stephensi* isofemale line (designated LB1) and the aposymbiotic *A. stephensi* line (*w*AlbB cured, designated LBT) were previously established (Bian et al., 2013). Aposymbiotic line LBT was derived from the LB1 strain after treatment with tetracycline (Bian et al., 2013). Approximately 4 years and 21 generations passed since that treatment was done, before this study was conducted. The presence or absence of *Wolbachia* in LB1 or LBT was confirmed by PCR of DNA extractions from abdomens using primers wsp81F (TGGTCC AATAAGTGATGAAGAAAC) and wsp691R (AAAAATTAAACGCTACTCCA). No *Wolbachia* infection was found in LBT individuals but all LB1 individuals were positive across the experimental groups (**Supplementary Figure S1**). Females of each strain were sampled 1 week after adult emergence, designated here as “LB1-SM” and “LBT-SM” given that they had sugar meals but no blood meals yet. Other females were provided a blood meal at 7 days after adult emergence by allowing them to feed on anesthetized BALB/c mice for 20 min. These mosquitoes were sampled 24 h after the blood meal, and were designated “LB1-BM” and “LBT-BM,” respectively. Finally, other blood fed females of the LB1 and LBT strains were sampled after an interval of 1 week following the blood meal as “aged” mosquito samples (15 days after adult emergence and 8 days after the blood meal), during which time they were provisioned 10% sugar solution. These mosquitoes were designated “LB1-PBM” and “LBT-PBM,” respectively.

### DNA Extraction, Library Construction, and 16S rRNA Sequencing

All DNA extractions were performed in a laminar flow biosafety cabinet to avoid contamination. Prior to dissection, mosquitoes were disinfected externally with 70% ethanol (three rinses), followed by a rinse with sterile water. Midguts were dissected with sterile forceps, and transferred to 200 μl of sterile phosphate-buffered saline (PBS) buffer. Midguts from six individuals were homogenized by crushing with a sterile pestle in the same tube as a single sample. The volume was re-suspended in 200 μl of lysis buffer and DNA extracted with the DNeasy Blood & Tissue Kit (Qiagen) following the manufacturer’s recommendations. Six biological replicates (total 36 midguts) were used for each of the six experimental treatments; a total of 216 mosquitoes were used in this study. The DNA concentration was measured using Qubit^TM^ dsDNA HS Assay Kits and the DNA integrity was tested by PCR using primers 63F (CAGGCCTAACACATGCAAGTC) and 1387R (CGGAACATGTGWGGCGGG). Amplicon tagging and sequencing were conducted at the Research Technology Support Facility (RTSF) at Michigan State University. DNA was amplified by using the primers 515f (GTG CCA GCM GCC GCG GTA A) and 806r (NNN NNN GGA CTA CHV GGG TWT CTA AT), which targets the V4 region of bacterial 16S rDNA. The reverse primer contains a 6-bp error-correcting barcode unique to each sample. The purified amplicons were pooled, loaded on an Illumina MiSeq flow cell, and sequenced in a 2 × 250 bp paired end format using a 500 cycle v2 reagent cartridge. Base calling was done by Illumina Real Time Analysis (RTA) v1.18.54 and output of the RTA was demultiplexed and converted to FastQ format by Illumina Bcl2fastq v1.8.4.

### DNA Sequence Analysis

Sequencing reads from the FastQ format were processed and analyzed using the Mothur package^1^ v.1.37.0. After denoising procedures by PyroNoise, Uchime, preclustering, good quality sequences (>250 bp) without detectable sequencing errors or chimeras and removing rare operational taxonomic units (OTUs; 5 or fewer) were used for assigning OTUs using an average neighbor algorithm (97% similarity cutoff). OTUs were classified at the genus level using the Bayesian method. When there were no references for the interested bacteria in the database used by Mothur, we retrieved the representative sequences for specific OTUs and submitted the sequences to GenBank (NCBI) for a BLAST search for purposes of classification. Data were further analyzed by first trimming sequence quality with different cutoffs using standard filtering tools (Schloss et al., 2009). Rarefaction curves were built to estimate sample coverage. Non-parametric estimates of richness were made using the Chao index and Abundance Coverage Estimator (cut-off threshold, 97%). Within-sample diversity (or α-diversity) was calculated using Simpson and Shannon diversity indices. Welch’s *t*-test was used to compare diversity indexes between *Wolbachia*-infected and *Wolbachia*-cured mosquitoes with different diets and at different rearing ages. Community composition (or β-diversity) was compared among treatments using Bray–Curtis dissimilarities. Differences in community composition among treatments were tested using permutational multivariate analysis of variance (PERMANOVA). Richness and α-diversity analyses were done in mothur. β-diversity analyses were done in R (v. 3.2.0). Bray–Curtis dissimilarity matrices were created using the vegdist() function (method = “bray”) and PERMANOVA tests (999 permutations) were done using the adonis() function in the vegan package [v. 2.3-2 (Oksanen et al., 2015)]. Principal coordinate analyses (PCoA) ordinations were created using the pcoa() function in the ape package [v. 3.4 (Paradis et al., 2004)].

### Nucleotide Sequence Accession Number

The raw sequences of this study have been deposited in the Sequence Read Archive (accession number: SRP072068).

## Results

### Characteristics of Midgut Bacteria Community Libraries

**FIGURE 1:**
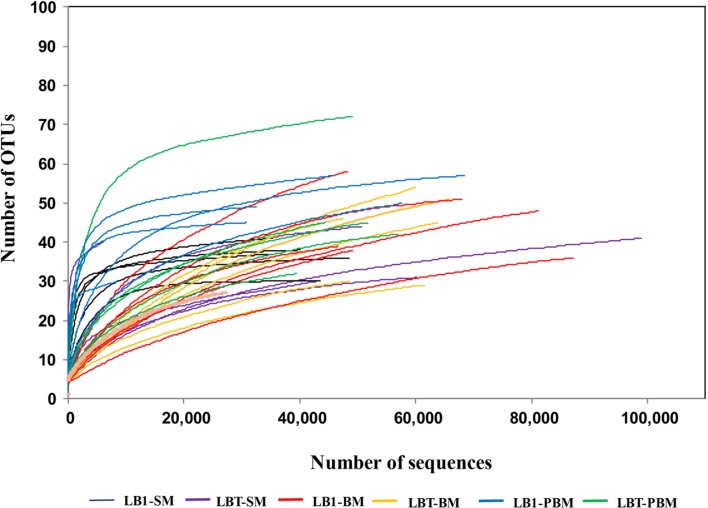
**Rarefaction analysis of bacterial 16S rRNA gene libraries from *Wolbachia*-infected and *Wolbachia-*cured *A. stephensi* samples that were sugar fed, blood fed, and post-blood fed.** Operational taxonomic units (OTUs) were grouped with a 97% similarity. LB1-SM, *Wolbachia*-infected mosquitoes with sugar meal; LBT-SM, *Wolbachia*-cured mosquitoes with sugar meal; LB1-BM, *Wolbachia*-infected mosquitoes with blood meal; LBT-BM, *Wolbachia-*cured mosquitoes with blood meal; LB1-PBM, post-blood fed *Wolbachia*-infected mosquitoes; LBT-PBM, post-blood fed *Wolbachia-*cured mosquitoes.

Approximately 2,130,826 sequence reads were successfully retained. After removal of short (<250 bp), possibly chimeras and rare OTUs (5 or fewer, see **Supplementary Table S1**), a total of 1,704,538 good quality sequences were available for further analysis (**Supplementary Table S2**). These sequences were assigned to 394 OTUs (**Supplementary Table S1**). Sequence number for each sample was normalized to the minimal readings (6,056) by randomly subsampling to minimize the biases generated by sequencing depth (**Supplementary Table S2**). The average Good’s coverage was 99.9% (**Supplementary Table S2**). The rarefaction curves (**Figure 1**) showed that the samples from some of these mosquito collections did not reach saturation to an asymptote, indicating that additional rare bacterial taxa likely occur in them, but all deflected as they rose with sampling effort in a characteristic concave function (**Figure 1**).

### Effects of *Wolbachia* Infection, Diet, and Age on Composition of the Gut Microbiota

**FIGURE 2:**
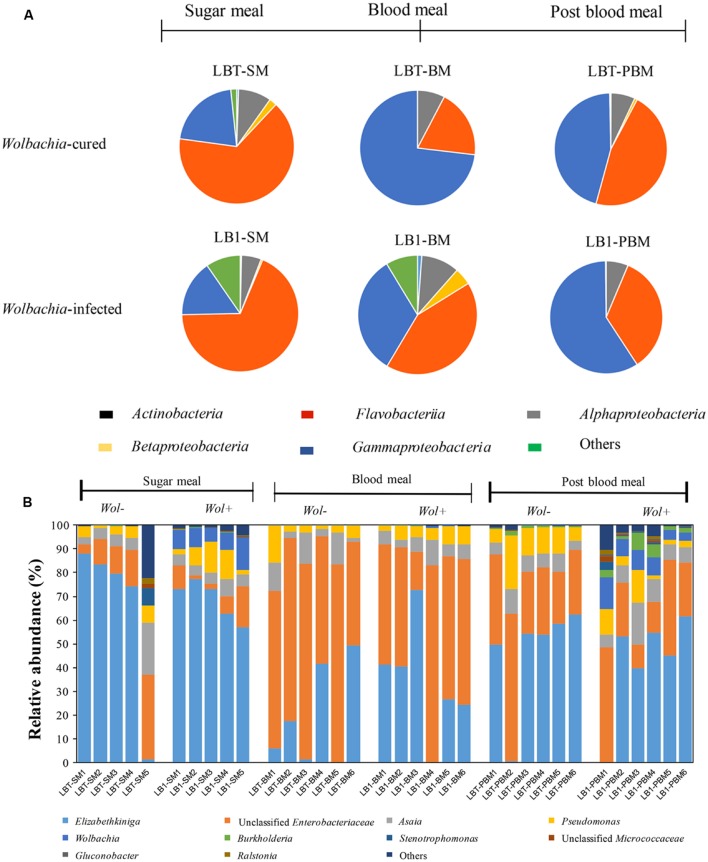
**Gut bacterial composition at class level and the relative abundance of the top 10 genera in *Wolbachia*-infected and *Wolbachia-*cured mosquito samples by dietary treatment.** **(A)** Taxonomic classification of bacterial reads retrieved from the *Wolbachia*-infected and *Wolbachia-*cured mosquitoes. **(B)** Relative abundance of 10 most abundant OTUs (97% similarity). Unclassified *Enterobacteriaceae* or *Micrococcaceae* represent the reads that were assigned to the family *Enterobacteraceae* or *Micrococcaceae*, but could not be assigned to a genus. *Wol*+, *Wolbachia*-infected mosquito; *Wol*-, *Wolbachia*-cured mosquito.

Operational taxonomic units were assigned to over 16 bacterial phyla and to unclassified bacteria (**Supplementary Table S3**). Single OTUs assigned to the classes *Flavobacteriia*, *Gammaproteobacteria*, and *Alphaproteobacteria* were most frequent, together accounting for more than 90% of the sequences, and were dominant in experimental treatments (**Figure 2A**). Sequences of bacteria in the classes *Actinobacteria* and *Betaproteobacteria* were also detected, but they were not uniformly represented in every sample. Some bacteria (2% of the total community) could not be assigned to a certain rank (unclassified), suggesting the existence of previously uncharacterized taxa. However, OTUs assigned to other classes were not abundant (i.e., <1%) and/or were absent from some samples or groups. The top 10 OTUs at the genus level comprised 99.2% of the total sequences (**Figure 2B**). The predominant OTUs were represented by *Elizabethkingia* (46.9%), unclassified *Enterobacteriaceae* (37.2%), *Asaia* (6.4%) and *Pseudomonas* (6.0%).

There was no significant difference in bacterial class abundance between *Wolbachia-*infected (LB1-SM) and *Wolbachia-*cured mosquitoes (LBT-SM) (Welch’s *t*-test) (**Figure 2A**). At the genus level, the most abundant OTU in the midgut of *A. stephensi* LBT (*Wolbachia-*cured) was *Elizabethkingia*, accounting for 65% of the community (**Figure 2B**). A similar proportion of *Elizabethkingia* was found in *A. stephensi* LB1 (69%), indicating that *Wolbachia* infection did not significantly alter its relative abundance (*p* > 0.05). Next to *Elizabethkingia*, unclassified *Enterobacteriaceae* were the second most abundant OTU in sugar-fed young *Anopheles* mosquitoes (LB1-SM and LBT-SM), which accounted for between 8 and 15% of the OTUs (**Figure 2B**). The number of *Enterobacteriaceae* OTUs was not statistically different between LB1-SM and LBT-SM (*p* > 0.05; **Figure 2B**). *Asaia* OTUs ranged in abundance from 5 to 8% of the total bacterial community (**Figure 2B**) in LB1-SM and LBT-SM, respectively, indicating that the colonization and persistence of *Asaia* in *A. stephensi* midguts occurred in a stable *Wolbachia* infection mosquito line as well as in *A. stephensi* without *Wolbachia* infection (*p* > 0.05; **Figure 2B**). Similar to *Asaia*, *Pseudomonas* OTUs were equally associated with both mosquito lines (*p* > 0.05; **Figure 2B**). As expected, the relative abundance of *Wolbachia* was 8.6% of the total bacterial OTUs in LB1-SM while it was nearly absent (<0.01% of OTUs) in LBT-BM (**Figure 2B**).

At 24 h after a blood meal, the relative abundance of *Elizabethkingia* decreased from 65 to 19% in LBT (*p* < 0.05) and decreased from 69 to 34% in LB1 mosquitoes, respectively (*p* < 0.05). By contrast, after a blood meal the *Enterobacteriaceae* OTUs increased 7.1 and 4.4-fold in LB1-BM and LBT-BM within 24 h (*p* < 0.05), respectively. Compared to sugar meals, the relative abundance of *Asaia* or *Pseudomonas* was not significantly changed by blood feeding (*p* > 0.05; **Figure 2B**). The relative abundance of *Wolbachia* in the midgut decreased to 0.18% of the total taxa in 24 h after blood meals (**Figure 2B**).

In mosquitoes at age 15 days (that is, 8 days post-blood meal), the frequency of *Elizabethkingia* OTUs represented 42% of the bacterial community in guts of *Wolbachia*-infected mosquitoes (LB1-PBM), showing that relative abundance of these OTUs in 15 days old LB1-PBM was significantly different from 7 days old LBT-SM mosquitoes (*p* < 0.05; **Figure 2B**). However, there was no statistical difference between LBT-SM and LBT-PBM mosquitoes (*p* > 0.05; **Figure 2B**). The relative abundance of *Enterobacteriaceae* OTUs in 15 days old LBT and LB1 mosquitoes was 2.2 and 3.4-fold higher than that in young LBT and LB1 mosquitoes (*p* < 0.05), respectively. The abundance of *Asaia* was consistent and not affected by age (*p* > 0.05), and accounted for 8.9 and 6.5% of bacterial community in older *Wolbachia*-infected and *Wolbachia-*cured mosquitoes, respectively. The abundance of *Pseudomonas* in older, *Wolbachia*-infected mosquitoes (LBT-PBM) was 2.6-fold higher than in young LBT mosquitoes (*p* < 0.05), while it remained stable between young and old, *Wolbachia-*cured mosquitoes (*p* > 0.05; **Figure 2B**). The relative abundance of *Wolbachia* OTUs in LB1-PBM reached up to 7.4% of total bacterial community level, which was comparable to that in LB1-SM (*p* > 0.05; **Figure 2B**).

### Alpha and Beta Bacterial Diversity in *Wolbachia*-Infected and *Wolbachia-*Cured Mosquitoes of Different Diets and Ages

To analyze differences in alpha diversity, we compared diversity (Shannon index) and richness (Chao1). There was no significant difference in Shannon diversity between LB1-SM and LBT-SM or LB1-BM and LBT-BM, showing that intracellular *Wolbachia* infection did not affect microbial diversity in young mosquito midguts (**Table 1**; **Supplementary Figure S2**). However, the Shannon index was different between LB1-PBM and LBT-PBM samples (*p* < 0.05), indicating that *Wolbachia* infection only changed gut microbial diversity in older mosquito. After blood feeding, the Shannon diversity in LB1-BM was not significantly different from that in LB1-SM (*p* > 0.05) and there was no significant difference between LBT-BM and LBT-SM (*p* > 0.05) (**Table 1**; **Supplementary Figure S2**). Thirdly, microbial diversity was significantly different between older mosquitoes (LBT-PBM) and young mosquitoes (LBT-SM) with *Wolbachia* infection (*p* < 0.05). However, in non-*Wolbachia* infection mosquitoes, the microbial diversity was similar between older mosquitoes (LB1-PBM) and young mosquitoes (LB1-SM) (p < 0.05; **Table 1**; **Supplementary Figure S2**). For community richness comparisons (Chao1), no significant differences were observed between *Wolbachia*-infected mosquitoes (LB1-SM, LB1-BM, or LB1-PBM) and *Wolbachia-*cured ones (LBT-SM, LBT-BM, or LBT-PBM) (p > 0.05; **Supplementary Figure S2**). However, blood-fed mosquitoes with *Wolbachia* infection had a significantly lower number of estimated OTUs (Chao1) than sugar-fed ones with *Wolbachia* infection (i.e., LB1-BM vs. LB1-SM, *p* < 0.05) while there was no significant difference between blood-fed and sugar-fed mosquitoes without *Wolbachia* infection (LBT-BM vs. LBT-SM, *p* > 0.05) as shown in **Supplementary Figure S2**, indicating that the blood meal decreased richness in *Wolbachia* infected mosquitoes. However, the richness (Chao1) in the guts of older mosquitoes (LB1-PBM or LBT-PBM) was not significantly different from that in of younger ones (LB1-BM or LBT-BM) after blooding feeding (*p* > 0.05; **Supplementary Figure S2**).

**Table 1:** Richness and diversity estimation of the 16S rRNA gene libraries.

Sample	Cutoffs	Simpson	Shannon	Chao	Ace
LBT-SM	0.03	0.58 ± 0.23	0.99 ± 0.71	32.15 ± 14.16	41.24 ± 14.35
LB1-SM	0.03	0.50 ± 0.01	1.13 ± 0.23	39.91 ± 10.31	41.23 ± 10.07
LBT-BM	0.03	0.57 ± 0.12	0.78 ± 0.19	39.81 ± 32.14	22.07 ± 25.94
LB1-BM	0.03	0.50 ± 0.11	0.92 ± 0.16	25.96 ± 11.81	40.49 ± 24.43
LBT-PBM	0.03	0.41 ± 0.04	1.12 ± 0.06	39.23 ± 13.51	64.59 ± 24.09
LB1-PBM	0.03	0.33 ± 0.07	1.56 ± 0.31	36.76 ± 10.51	37.21 ± 9.64


**FIGURE 3:**
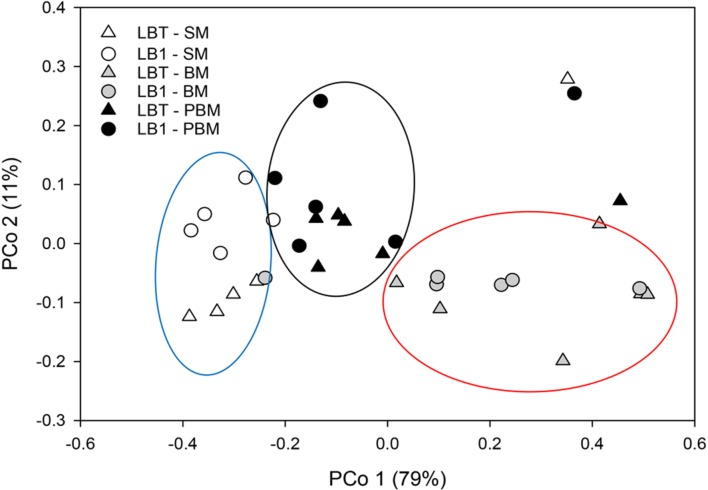
**Principal coordinate analysis of bacterial the community structure using Bray–Curtis distances.** Symbols represent one mosquito collection (six pooled mosquito guts). Distances between symbols on the ordination plot reflect relative dissimilarities in community structures.

For beta diversity analysis, we examined the relationships in gut microbiota between different diet, age and *Wolbachia* infection status by using principal coordinate analyses (PCoA) based on Bray–Curtis distance (**Figure 3**). The first two components captured 90% of the variance among samples. Community composition differed among all three diet types (PERMANOVA; *F* = 10.93, *p* < 0.05, **Table 2**). Bacterial communities from the mosquitoes fed on sugar separated from those fed on blood and those PBM along PC1, which explained 79% of the variation (**Table 3**). However, community composition was similar between *Wolbachia*-infected and *Wolbachia-*cured groups across all diet treatments (PERMANOVA; *F* = 2.86, *p* > 0.05, **Table 2**).

**Table 2:** Pseudo *F* table of PERMANOVA analysis based on Bray–Curtis dissimilarities.

Source of variance	Sum of squares	Degrees of freedom	Mean square	*F*	*R*^2^	*p*^∗^
*Wolbachia*	0.186	1	0.186	2.861	0.053	0.086
Diet	1.418	2	0.709	10.931	0.404	**0.001**
*Wolbachia*^∗^Diet	0.088	2	0.044	0.681	0.025	0.549
Residuals	1.816	28	0.065		0.518	
Total	3.509	33			1	


*^∗^OTUs with 5 or fewer sequences were excluded. Significant *p*-values (≤0.05) are bolded.*

**Table 3:** Pseudo *F* table of pairwise comparisons of diet types using PERMANOVA.

Pairwise comparison	Source of variance	Sum of squares	Degrees of freedom	Mean square	*F*	*R*^2^	*p*^∗^
SM vs. BM	Diet	1.314	1	1.314	19.660	0.496	**0.001**
	Residuals	1.337	20	0.067		0.504	
	Total	2.652	21			1	
SM vs. PBM	Diet	0.308	1	0.308	4.261	0.176	**0.021**
	Residuals	1.444	20	0.072		0.824	
	Total	1.752	21			1	
BM vs. PBM	Diet	0.522	1	0.522	8.208	0.272	**0.004**
	Residuals	1.399	22	0.064		0.728	
	Total	1.921	23			1	


*^∗^OTUs with 5 or fewer sequences were excluded. Significant *p*-values (≤0.05) are bolded.*

## Discussion

The bacteria (i.e., microbiota) associated with mosquitoes have significant roles throughout the mosquito lifecycle, in providing energy and nutrients, and regulating development, fecundity, and immune responses (Sinkins, 2004; Chouaia et al., 2012; Pan et al., 2012; Coon et al., 2014). These symbiotic bacteria find shelter and nutrients within this environment and could properly be referred to as commensals for these beneficial reasons, even if the beneficial associations of the bacteria to mosquitoes are not entirely clear. Stable, intracellular *Wolbachia* infection in *A. stephensi* (LB1) was achieved previously through embryonic inoculation and multi-generational, vertical passage (Bian et al., 2013). It was done without perturbation of the natural microbiota (as shown here) and without excessive mortality after blood meals (Bian et al., 2013; Joshi et al., 2014). More recently and by contrast, vertical persistence of *Wolbachia* infection in *A. stephensi* was achieved only when symbiotic bacteria were perturbed by antibiotics (Hughes et al., 2014). In that same study, a combination of *Wolbachia* and *Asaia* caused mosquito mortality after a blood meal, a finding suggestive of a physiologic incompatibility between *Wolbachia* infection and presence of a constitutive microflora when the stressors of the blood meal arrive (Hughes et al., 2014). Indeed, if such an incompatibility exists, it would obviate the possibility of a dual, anti-malaria parasite strategy based on immune-mediating effects of *Wolbachia* infection and paratransgenesis of select members of that microflora. This possibility, in part, motivated the research presented here.

The similarity of the gut bacterial community structure, as measured by composition of taxa as well as by α- and β-diversity, between *Wolbachia*-infected LB1 and *Wolbachia-*cured LBT mosquitoes indicates that there were no significant *Wolbachia*-mediated effects on the structure of the commensal bacterial community; and that the bacterial assembly was resilient to any effects, such as innate immune effects, that the presence of *Wolbachia* might impose. Therefore, coexistence of these gut bacteria with *Wolbachia* was not problematic in the stable *Wolbachia*-infected *Anopheles* progeny. These findings contrast with other studies demonstrating very strongly negative effects of combination of gut bacteria and *Wolbachia* on host fitness (high mortality rate after a blood meal), particularly due to *Asaia* bacteria (Hughes et al., 2014). One explanation for the difference is in the way the *Wolbachia* infection was originally introduced into the mosquitoes: embryonic microinjection followed by stable, *trans*-generational perpetuation of infection (Bian et al., 2013); or intrathoracic inoculation without perpetuation (Hughes et al., 2014). The latter likely led to a larger and more acute dose of the bacteria, than one inherited in the germ line. However, the detailed mechanisms need to be further investigated.

The blood meal profoundly affects bacterial community composition in the midgut of several mosquito species, including *A. gambiae*, *A. stephensi*, and *A. coluzzii* (Wang et al., 2011; Gimonneau et al., 2014; Tchioffo et al., 2016). The main influencing factors included dramatic changes in mosquito gut temperature and appearance of oxidative stress (Luckhart et al., 1998; Peterson and Luckhart, 2006; Benoit et al., 2011). Especially, the ingested red blood cells from animals carry an enormous amount of hemoglobin, which causes a massive release of heme in the midgut, thus leading to a dramatic change in gut conditions (Wang et al., 2011; Tchioffo et al., 2016). Blood meals induce midgut epithelia to produce nitric oxide which is also a source for free radicals (Luckhart et al., 1998). Hematophagous mosquitoes exhibit detoxification mechanisms in response to these free radicals, but the role of commensal bacteria in the gut in that same process and how those bacteria survive them are not known (Peterson and Luckhart, 2006; Benoit et al., 2011). After blood meals, *Enterobacteriaceae* dramatically increased, which was consistent with those reported in *A. gambiae* (Wang et al., 2011). Wang et al. (2011) found several stress response systems existing in the *Enterobacteriaceae* genomes. Possibly, this group of bacteria interacts with the mosquito host in response to the oxidative stressors present in the blood meal bolus (Wang et al., 2011). Therefore, blood-induced mortality in transiently *Wolbachia*-infected *Anopheles* mosquito could be caused by a combinative effect, that is, innate immune response elicited by *Wolbachia* infection and by dysregulated microbiota (Luckhart et al., 1998; Pan et al., 2012; Hughes et al., 2014). Both of them caused an unusual high level of active radicals in midgut, which can cause destructive damage to mosquitoes (Luckhart et al., 1998). Although there was a dramatic change in microbial structure after blood meals (compared to sugar meals), the core microbial compositions and community structure between *Wolbachia*-infected and *Wolbachia-*cured mosquitoes were rather similar (**Figure 3**).

When switching diet from blood to sugar meals and aging the insects, we found that the microbial community structure was different between the young (before blood meal) and aged mosquitoes (post-blood meal) in both mosquito lines. However, the relative abundance of transiently increased bacteria such as *Enterobacteriaceae* decreased when blood digestion was complete; the dominant *Elizabethkingia* abundance at age 15 days, and after the blood meal, was similar to before the blood meal (**Figure 2B**). The dominant bacterial community patterns we observed here were similar to those reported in *A. gambiae* (Wang et al., 2011). Collectively, *Asaia*, *Elizabethkingia*, and *Enterobacteriaceae* were present and predominant in aged, blood-fed, *Wolbachia*-infected *A. stephensi*, providing the possibility to devise an efficient malaria control reagent based on a combination of effects of *Wolbachia* and effects of paratransgenic gut microbiota (Federici et al., 2003; Favia et al., 2008; Cirimotich et al., 2011; Boissière et al., 2012; Capone et al., 2013; Bongio and Lampe, 2015; Chen et al., 2015).

Previous studies demonstrated that *Wolbachia* infected the midgut in *A. stephensi* and *A. gambiae* mosquitoes though its distribution level was much lower than that in the fat body (Bian et al., 2013). After a blood meal, the dramatic decrease of *Wolbachia* OTUs in LB1 gut was consistent with a previous observation by Hughes et al. (2014). Several factors including heme release, ROS level change, and interactions with gut bacteria contributed to this change (Pan et al., 2012; Bian et al., 2013; Hughes et al., 2014). However, after removing gut bacteria by antibiotic treatment (especially *Asaia*), *Wolbachia* persisted at a level similar to that non-blood fed mosquitoes, indicating gut microbiota suppressed *Wolbachia* level after a blood meal (Hughes et al., 2014).

Operational taxonomic units of *Asaia* (up to 8.9% of the total taxa) were consistently detected in *Wolbachia*-infected *A. stephensi* guts here. The relative abundance of *Asaia* in LBT or LB1 mosquitoes was similar regardless of *Wolbachia* infection, diet switching and rearing ages. This observation differs from the study conducted by Hughes et al. (2014) where *Asaia* concentration was much higher in blood fed mosquitoes than in non-blood fed ones. The initial assembly of the gut microbiota might account for this discrepancy: *Asaia*, *Pseudomonas*, and unclassified *Gammaproteobacteria* were the most predominant bacteria (Hughes et al., 2014). *Asaia* is a common commensal of several mosquito species such as *Aedes*, *Anopheles*, and *Culex* (Favia et al., 2008; Rossi et al., 2015). Its physiological roles in mosquitoes have been suggested to be nutrient scavenging and regulation of larval development (Chouaia et al., 2012). Recent studies showed antagonism between *Asaia* and *Wolbachia* in the reproduction organs (such as ovary; Rossi et al., 2015). Further, antagonistic interactions between *Wolbachia* and the gut microbiota, particularly *Asaia*, resulted in significant fitness costs in the mosquito host (Hughes et al., 2014). Our observation here showed that *Asaia* was stably maintained in both LB1 and LBT guts, which was consistent with Rossi et al. (Rossi et al., 2015).

The relative abundance of *Elizabethkingia* OTUs accounted for more than 60% of the total microbial taxa, highlighting their importance in *Anopheles* mosquitoes. Presumably, they contribute to metabolism of sugars in the gut environment, as evidenced by the SusC/SusD-like polysaccharide transport system(s) and glycosidases in their genome (Kukutla et al., 2014). Supplementation of *Elizabethkingia* cells in the sugar meal for *A. stephensi* led to approximately 50% more egg production compared to those without supplementation, suggesting that *Elizabethkingia* contributed to animal erythrocyte lysis and thus increased host’s fecundity in mosquitoes (S. Chen, unpublished). *Elizabethkingia* was frequently detected in water, sediments and insects including field caught, semi-natural reared, and insectary reared mosquitoes, showing that it adapted to diverse ecological niches in nature, but it is commonly found in *Anopheles* midguts (Chen et al., 2015). Pyrosequencing analysis showed that *Elizabethkingia* OTUs were more abundant in larval *A. gambiae* mosquitoes than in the aquatic medium in which they were being reared, but were present in the latter suggesting a common environmental source (Wang et al., 2011). Furthermore, *Elizabethkingia anophelis* and its nearly identical taxon, *Elizabethkingia meningoseptica*, were among the most predominant bacteria in adult *A. gambiae* and *A. stephensi* (Wang et al., 2011; Akhouayri et al., 2013; Ngwa et al., 2013).

## Author Contributions

SC and EW wrote the manuscript. DJ and SC performed the experiments. SC, JZ, ZX, BN, and EW performed the data analysis.

## Conflict of Interest Statement

The authors declare that the research was conducted in the absence of any commercial or financial relationships that could be construed as a potential conflict of interest.

The reviewer HS and handling Editor declared their shared affiliation, and the handling Editor states that the process nevertheless met the standards of a fair and objective review.

## Footnotes

1 http://www.mothur.org
