# Supplementary material for: Persistent Infection by Wolbachia wAlbB Has No Effect on Composition of the Gut Microbiota in Adult Female Anopheles stephensi
Source: Front Microbiol. 2016 Sep 21;7:1485. doi: 10.3389/fmicb.2016.01485 (PMC5030273; doi:10.3389/fmicb.2016.01485)
Supplement: Supplementary file 1 [file Data_Sheet_1.PDF]

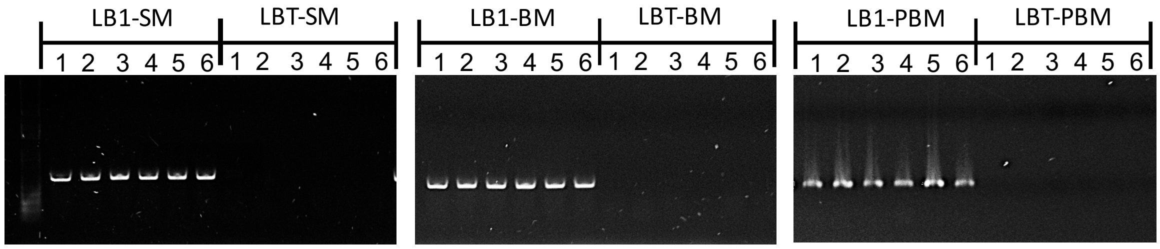

FIGURE S1. *Wolbachia* presence and absence in LB1 and LBT confirmed by PCR. The primers used were described in Bian et al (2013). LB1 refers to an *A. stephensi* strain with stable *Wolbachia* infection. LBT refers to a strain of *A. stephensi* derived from LB1 but which was cured of *Wolbachia* infection by tetracycline treatment. SM, sugar meal. BM, blood meal. PBM, post-blood meal.

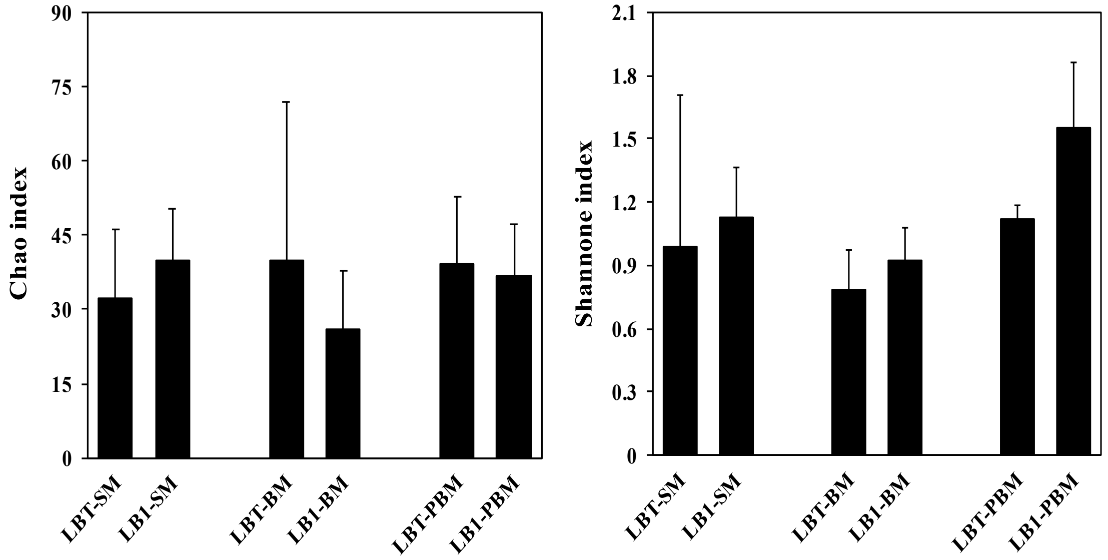

FIGURE S2. Comparison of Chao1 and Shannone index. Left panel, comparison of Chao1 index invarious mosquitoes. Right panel, comparison of Shannone index in various mosquitoes.

Table S1 Insert here.

Table S2 Summary of 16S rRNA sequencing tags for *Wolbachia*-infected and *Wolbachia*-cured mosquitoes with different diets and rearing ages (cut-off threshold, 97%).

Group	Cutoff	Readings	Coverage	Observed OTUs	Invsimpson	Shannoneven
LBT-SM1	0.03	50,645	0.9983	23	1.28	0.17
LBT-SM2	0.03	98,798	0.9992	10	1.41	0.26
LBT-SM3	0.03	60,415	0.9988	14	1.54	0.28
LBT-SM4	0.03	43,046	0.9992	18	1.73	0.29
LBT-SM5	0.03	6,056	0.9990	40	5.21	0.61
LB1-SM1	0.03	35,501	0.9988	33	1.80	0.29
LB1-SM2	0.03	43,782	0.9980	25	1.63	0.27
LB1-SM3	0.03	48,793	0.9985	32	1.80	0.28
LB1-SM4	0.03	40,014	0.9992	33	2.36	0.38
LB1-SM5	0.03	35,318	0.9997	32	2.65	0.41
LBT-BM1	0.03	47,474	0.9983	14	2.08	0.38
LBT-BM2	0.03	63,904	0.9993	10	1.60	0.31
LBT-BM3	0.03	60,108	0.9983	15	1.44	0.23
LBT-BM4	0.03	66,017	0.9979	17	2.17	0.31
LBT-BM5	0.03	48,642	0.9990	13	1.40	0.21
LBT-BM6	0.03	61,432	0.9992	13	2.29	0.37
LB1-BM1	0.03	87,148	0.9995	8	2.33	0.47
LB1-BM2	0.03	81,331	0.9992	11	2.38	0.42
LB1-BM3	0.03	46,793	0.9985	18	1.79	0.30
LB1-BM4	0.03	48,294	0.9983	22	1.42	0.20
LB1-BM5	0.03	49,385	0.9983	18	2.25	0.36
LB1-BM6	0.03	68,065	0.9987	15	2.24	0.38
LBT-PBM1	0.03	44,576	0.9983	23	2.52	0.36
LBT-PBM2	0.03	49,248	0.9972	54	2.22	0.29
LBT-PBM3	0.03	39,452	0.9985	17	2.63	0.42
LBT-PBM4	0.03	27,222	0.9988	14	2.59	0.44
LBT-PBM5	0.03	57,075	0.9988	14	2.45	0.43
LBT-PBM6	0.03	51,964	0.9982	22	2.14	0.32
LB1-PBM1	0.03	8,603	0.9997	28	3.70	0.58
LB1-PBM2	0.03	45,724	0.9987	48	2.89	0.39
LB1-PBM3	0.03	32,753	0.9997	41	4.33	0.48
LB1-PBM4	0.03	30,886	0.9993	40	3.00	0.46
LB1-PBM5	0.03	57,594	0.9987	22	2.68	0.39
LB1-PBM6	0.03	68,480	0.9992	25	2.28	0.37

Table S3 Gut bacterial composition at the phylum level of the microbiota associated with *Wolbachia*-infected and *Wolbachia*-cured mosquitoes

Phyla	LBT-SM	LB1-SM	LBT-BM	LB1-BM	LBT-PBM	LB1-PBM
Bacteroidetes	65.308	68.561	19.214	34.460	46.556	42.509
Proteobacteria	32.903	21.679	80.747	65.332	53.195	47.922
Actinobacteria	0.512	0.363	0.011	0.012	0.071	1.151
Firmicutes	0.032	0.048	0.004	0.012	0.026	0.051
OD1	0.396	0.073	0.002	0.001	0.005	0.065
Verrucomicrobia	0.153	0.006	0.000	0.012	0.003	0.004
Chlamydiae	0.072	0.003	0.000	0.000	0.004	0.000
Armatimonadetes	0.000	0.000	0.000	0.000	0.002	0.000
Deinococcus-Thermus	0.000	0.000	0.000	0.000	0.001	0.000
Gemmatimonadetes	0.003	0.000	0.000	0.000	0.001	0.000
Planctomycetes	0.020	0.001	0.000	0.000	0.000	0.000
Cyanobacteria	0.005	0.055	0.006	0.000	0.016	0.137
OP11	0.001	0.000	0.000	0.003	0.000	0.032
SR1	0.000	0.058	0.000	0.000	0.000	0.000
Acidobacteria	0.003	0.051	0.001	0.002	0.003	0.023
Others	0.593	9.102	0.016	0.167	0.129	8.107
